# A Putative Prohibitin-Calcium Nexus in *β*-Cell Mitochondria and Diabetes

**DOI:** 10.1155/2020/7814628

**Published:** 2020-10-08

**Authors:** Gaurav Verma, Aparna Dixit, Craig S. Nunemaker

**Affiliations:** ^1^Molecular Metabolism, Lund University Diabetes Centre, Malmö -21428, Sweden; ^2^School of Biotechnology, Jawaharlal Nehru University, -110067, New Delhi, India; ^3^HCOM-Biomedical Sciences, Ohio University, Athens Camp, US-45701 Ohio, USA

## Abstract

The role of mitochondria in apoptosis is well known; however, the mechanisms linking mitochondria to the proapoptotic effects of proinflammatory cytokines, hyperglycemia, and glucolipotoxicity are not completely understood. Complex Ca^2+^ signaling has emerged as a critical contributor to these proapoptotic effects and has gained significant attention in regulating the signaling processes of mitochondria. In pancreatic *β*-cells, Ca^2+^ plays an active role in *β*-cell function and survival. Prohibitin (PHB), a mitochondrial chaperone, is actively involved in maintaining the architecture of mitochondria. However, its possible interaction with Ca^2+^-activated signaling pathways has not been explored. The present review aims to examine potential crosstalk between Ca^2+^ signaling and PHB function in pancreatic *β*-cells. Moreover, this review will focus on the effects of cytokines and glucolipotoxicity on Ca^2+^ signaling and its possible interaction with PHB. Improved understanding of this important mitochondrial protein may aid in the design of more targeted drugs to identify specific pathways involved with stress-induced dysfunction in the *β*-cell.

## 1. Prohibitin: Role in Mitochondrial Function and *β*-Cell Physiology

Mitochondria play a central role in pancreatic *β*-cell physiology by coupling glucose to insulin exocytosis [1]. However, mitochondria have emerged as critical players in Ca^2+^ induced *β*-cell death in the context of diabetes [2–5]. Ca^2+^ has a permissive role in apoptosis but a detailed mechanism of Ca^2+^-induced apoptosis is not yet defined. Sequestration of Ca^2+^ by the endoplasmic reticulum (ER) prevents high cytosolic Ca^2+^ (Ca^2+^)_c_ concentrations. Ca^2+^ efflux out of the ER can be induced by stresses such as cytokines and glucolipotoxicity [6–8]. High (Ca^2+^)_c_ can cause mitochondrial dysfunction, which may be mediated via prohibitin (PHB), a mitochondrial scaffold protein. Defects in ER Ca^2+^ storage and subsequent mitochondrial dysfunction are associated with the development of diseases like diabetes. It has also been shown that glycogen synthase kinase 3 beta (GSK3*β*) mediated Presenilin-1 phosphorylation is responsible for the ER Ca^2+^ leak in INS-1 *β*-cell that affects the mitochondrial functions [9].

PHBs are mitochondrial chaperone proteins recognized for their role in maintaining mitochondrial integrity [10]. Located in the inner mitochondrial membrane, two PHB isoforms, PHB1 and PHB2, form heterodimers to regulate mitochondrial structure and function [11–13]. Investigations with PHBs have established that it plays a crucial role in mitochondrial function in various complex disorders involving *β*-cells dysfunction and diabetes [14, 15]. However, the absence of PHBs could also induce the disintegration of mitochondria in nearly all kinds of cells that may lead to apoptosis. The mechanism by which PHB protects against mitochondrial-mediated cell death is not yet understood. As the role of PHB in Ca^2+^ homeostasis remains to be established, it is still unknown whether PHB function is mediated by Ca^2+^ signaling which is central to both normal and pathological cellular processes. In this review, we highlight the possible intricate tethering between PHB and Ca^2+^ signaling, and its role in deciding the fate of pancreatic *β*-cells. We discuss different modern approaches that can be used to study the role of Ca^2+^ signaling in PHB function. We also discuss mitochondrial alterations attributed to the PHB complex with a focus on its implications in Ca^2+^ regulation and disease pathophysiology. Finally, we have proposed a hypothesized function of PHB in Ca^2+^ regulation and its involvement in disease pathophysiology.

## 2. A Multifunctional Mitochondrial Protein?

The eukaryotic mitochondrial PHB complex consists of two heterodimer subunits, PHB1, and PHB2 [10, 12, 16]. PHB1, the principal mammalian PHB, is a potential tumor suppressor due to its antiproliferative action, and therefore named prohibitin [17]. Subsequently, this antiproliferative action was attributed to the 3′-UTR of the mRNA encoding PHB, [18]. PHB2 was characterized by virtue of its dimerization with PHB1.

PHB1 and PHB2, with molecular weights of 32 and 34 kDa, respectively, participate to form a ring-like macromolecular structure of 1 MDa at the mitochondrial inner membrane (IM). This high subatomic weight complex has been found in yeast, *C. elegans*, and mammals [19]. Considering the role of PHB in aging and cancer, assessing their possible role in these processes would be beneficial [20]. The PHBs can form both homodimers as well as heterodimers [21]. The homodimeric interaction of PHBs was not revealed but recent work by Yoshinaka et al. has revealed the PHB2 homodimer with the help of crystal structure that folds into an elongated shape with a highly charged surface. These homodimers exhibited three acidic residues, namely glutamates (E^229^, E^231^, and E^233^), and a glutamine (Q^227^) residue. Substitutions of these residues had no effect on the folding properties and physical hydrophobic interaction between PHBs homodimers but may be instrumental in the formation of mitochondrial interactome [22]. Similar studies have shown evidence of the formation of PHB homodimer in HeLa cells; however, the functional validation of these topology interactions demands further investigations [23]. On contrary, the participation of the PHB heterodimers in various cellular processes is known, but the mechanistic insights are not yet characterized. Having said that PHB1 and PHB2 function as the same heterodimeric complex in mitochondria, it is intriguing that they have differences in their phenotypes [24]. Depletion of either of the subunits results in dissociation of the complex [19]. Around 12 to 16 PHB heterodimers assemble to form a ring-like structure (20-25 nm) in diameter at the mitochondrial (IM) [25]. Extensive research in the field of mitochondrial biology has revealed a significant role of PHB in various cellular processes. The PHB complex is integrated into the mitochondrial inner membrane matrix through *N*-terminal hydrophobic domain patches. The PHB complex controls mitochondrial membrane protein alteration by m-AAA protease, which works as a holdase or unfoldase chaperone, and facilitates the folding of unfolded membrane proteins. The complex may also play a role in maintaining the mitochondrial genome. PHB complex also assists in mitochondrial morphogenesis by providing a scaffold that recruits membrane proteins to a designated lipid environment.

## 3. Diverse Role of Prohibitin in Transcriptional Regulation in Nucleus

**Figure 1:**
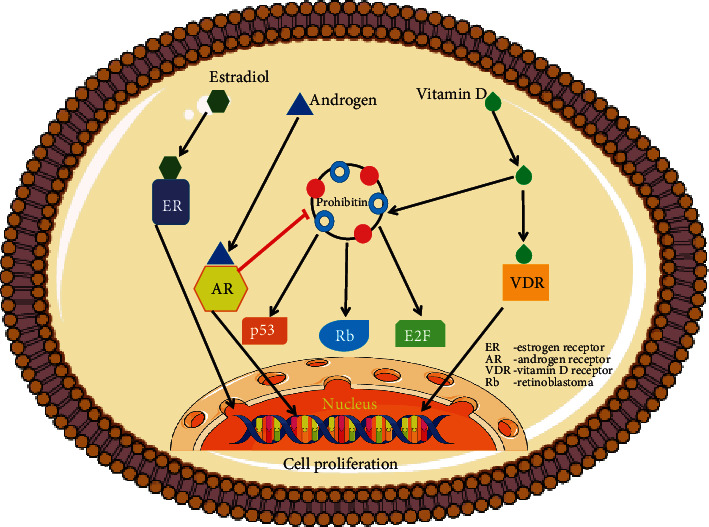
Prohibitins as gene expression regulators of the cell. Prohibitin complex interact with nuclear receptors, like ER, AR, VDR, and transcription factors p53, Rb, and E2F thereby regulate gene expression and cell proliferation of the target cell.

The transcription factor from the E2F family is involved in various biological processes including cell differentiation, proliferation, and apoptosis. It has been shown that PHBs inhibit the E2F transcriptional activity and regulate the cell cycle expression, transcription factors, and nuclear receptors [26, 27]. However, a detailed mechanism by which these interactions bring about the transcriptional regulatory activity is not yet elucidated. Interactions between PHBs and minichromosome maintenance complex of proteins (MCM2-7) have been shown [28]. Wang et al. [26] have also demonstrated that PHB1 interacts with the cell cycle retinoblastoma proteins (Rb), which are known to inhibit replication in S phase through the attenuation of PCNA function. The Rb-restricting region and another domain mapped to the *C*-terminal part of PHB are responsible for the suppression of E2F action. PHB interacts with nuclear p53 to mediate transcription of p53 target genes [29]. In addition, PHB suppresses the activity of transcription factors like E2F1 to E2F5, possibly via the Src activation, and thereby regulates cell proliferation [29]. Mutations present in the 3′-untranslated region of PHB in breast cancer cells suggest PHB transcriptionally regulates genes involved in breast cancer development [30]. In addition, PHB2 interacts with the transcription factor family of MyoD and myocyte enhancer element 2 (MEF2), potentially with the help of coactivators such as histone deacetylases HDAC1 [31]. It has been shown that PHB2 can inhibit muscle differentiation by repressing the transcriptional activity of both MyoD and MEF2. Interestingly, the coexpression of Akt and PHB2 has been shown to prevent the binding of PHB2 to MyoD. This stimulates muscle differentiation, suggesting that PHB2 may act as a myogenic repressor. Furthermore, PHB specifically binds to the estrogen receptor (ER) with the help of its ligand estradiol, resulting in suppression of ER transcriptional activity [32]. In addition to the ER-binding motif LXXLL near the *N*-terminus, amino acid residues spanning 175-198 in PHBs are required for its interaction with ER [33]. Knockdown of PHB by siRNA blocked the growth inhibitory effect of antiestrogens in MCF-7 breast cancer cells. PHB appeared to suppress androgen receptor-mediated translation and androgen-dependent cell development. These data suggest that PHBs might play important role in determining the sensitivity of estrogen target cells including breast cancer cells. Finally, a binding domain site for the Vitamin D receptor (VDR) has also been recognized in the promoter region of PHB1 [34]. VDR signaling is very important for the chemoprevention of breast cancer cells using Vitamin D analogs. However, the antiproliferative effects of Vitamin D in breast cancer cells are well recognized, but the target genes involved in this process are yet to be identified. Recent reports have suggested that PHB is a Vitamin D target gene and the Vitamin D treatment results in increased cellular PHB1 levels [35]. VDR/RXR binding sites are found in the promoter region of PHB gene which makes it a novel Vitamin D target gene involved in the antiproliferative activity. The immense variety and diversity of important nuclear binding partners demonstrate the breadth of PHB function and highlight its action in essential cellular physiology. The main function of the PHB complex is to control proteins involved in the cell proliferation and development, rather than binding to DNA as a transcription factor [36, 37]. All these reports suggest a critical function of PHBs in nuclear transcriptional regulation. Multiple roles of PHB in the nucleus are schematically depicted in (Figure 1).

## 4. Multifaceted Role of Prohibitin in Cell Survival and Apoptosis

Apoptosis, a controlled process of cell death, protects the organism from malfunctioning cells.

**Figure 2:**
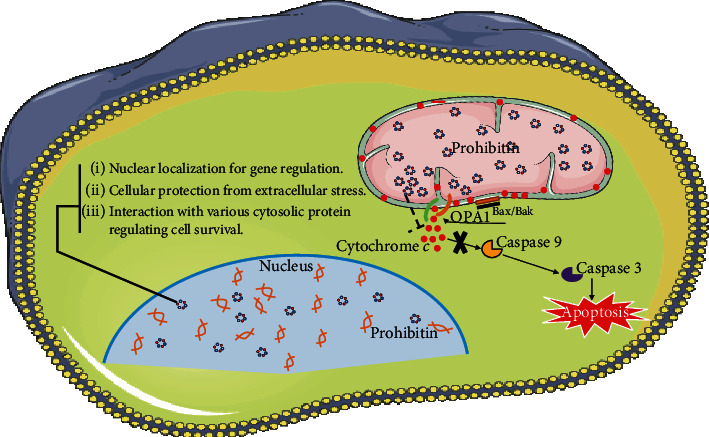
Putative role of prohibitin in mitochondria and nucleus. As mitochondrial chaperones, PHBs preserve the integrity of mitochondria. Their translocation to nucleus promotes binding of nuclear receptors and exhibits their action in the nucleus. PHBs interact with mitochondrial regulators including the key players of apoptotic pathways. PHBs interact with OPA1 complex (green and orange) which is located at the mitochondrial cristae junctional openings. Remodelling of these cristae majorly by OPA1 complex proteolysis or intermembrane proteases releases the cytochrome *c* in the cytosol. OPA1-mediated cristae opening is Bax/Bak dependent which is required for the cytochrome *c* release. Cytochrome *c* further activates the downstream effector molecules like caspase 9 and caspase 3 that culminates into apoptosis. Our hypothesized model proposes that overexpression of PHB could stabilize the mitochondrial structure and morphology that in turn may prevent the release of cytochrome *c*, activation of caspase, and apoptosis.

Apoptosis may be triggered by different stimuli such as proinflammatory cytokines, UV irradiation, reactive oxygen species (ROS), hormones, and growth factors. Mitochondria play an important role in mediating apoptosis through various signal transduction pathways. As previously mentioned, PHB is a membrane protein with different cellular localizations, and these differences in subcellular localization of PHB may direct what function it plays in the cell. A schematic diagram depicting the subcellular localization of PHB and its multiple functions in regulating cell fate is shown in (Figure 2).

Under stress conditions, PHB translocates to the nucleus and to the mitochondria, where it helps stabilizing the mitochondrial genome and controls mitochondrial morphology, biogenesis, and the intrinsic apoptotic pathway [38]. Differential expression of PHB within cells has a protective mechanism, which prevents the cells from death induced by the chemotherapeutic drug such as camptothecin [39]. Overexpression of PHB in B-cell lymphoma Ramos cells showed a significant decrease in camptothecin-induced cell death compared to parental cells. Levels of Rb family members that interact with E2F were decreased due to caspase-mediated degradation during camptothecin treatment. However, overexpression of PHB protected cells from cell death, suggesting an anti-apoptotic role. In leukemic cell lines, PHB acts as a nuclear protein substrate [40]. Camptothecin treatment resulted in translocation of PHB out of the nucleus to perinuclear regions [41]. The role of PHBs in cancer is complex. Some types of tumor overexpress PHB1 in the nucleus and some in mitochondria, whereas some of them do not express it at all. Given the existing evidence, fine-tuning of the PHB localization may be beneficial in selectively inducing apoptosis in cancer cells.

PHB acts as a stabilizer of inner mitochondrial membrane proteins. Therefore, loss of PHB severely affects the integrity of the mitochondria, as was shown for the first time in body wall muscle cells of *C. elegans*. The mitochondrial PHB complex is essential for embryonic viability and germline function in *C*. *elegans*. Loss of PHB results in mitochondria that appear disintegrated and fragmented [42]. These aspects of mitochondrial fragmentation in PHB deficient cells could be explained by the involvement of OPA1, which regulates the fusion process in mitochondria [36]. Considering PHB plays a crucial role in mitochondrial integrity and architecture, it is plausible that PHB could also participate in mitochondrial DNA (mtDNA) maintenance. This notion is supported by the siRNA-mediated knockdown of PHB1 in HeLa cells resulting in reduced mtDNA copy number, which is largely regulated by mitochondrial transcription factor A (TFAM) [43, 44]. PHB also correlates with oxidative stress and affects electron transport chain complex activity. However, the role PHB plays in regulating ROS production is currently not understood. The binding of PHB to mtDNA could explain these phenomena given mtDNA encodes oxidative phosphorylation (OXPHOS) subunits. PHBs play an important role in mitochondrial membrane integrity; therefore, it is possible that depletion of PHB could result in deficient OXPHOS activity.

Oxidative stress has been the main cause in the manifestation of various complex disorders including myocardial injury, diabetes, and neurodegenerative disorders [45–47]. Oxidative stress in aging mitochondria also leads to the accumulation of superoxide, which has been attributed as one of the major causes of mitochondrial dysfunction. Generation of mitochondrial ROS (mtROS) in T2D could be manifested by various mechanisms in which one of them is mitochondrial membrane potential (*∆Ψ*m). PHBs along with a mitochondrial anion carrier protein uncoupling protein 2 (UCP2) reduces this *∆Ψ*m and regulate the levels of mtROS and could be used as a biomarker surrogate for vascular health in patients with and without T2DM [48]. This PHB-UCP2 axis could also be one of the potential biomarkers that need to be investigated and further extrapolated as a disease biomarker. Having said that, PHB harbors diverse arrays of signaling, and its potential as therapeutics could be attributed by investigating its interacting partners like (Ca^2+^, ROS, and ATP) in a particular disease state. Furthermore, superoxide activates UCP2 that leaks the apoptosis-triggering protein cytochrome C from the inner mitochondrial membrane space when activated [49]. While the role of PHB as a mitochondrial chaperone has been characterized [50, 51], its role in oxidative stress has not been elucidated. PHB levels are elevated during oxidative stress, and overexpression of PHB significantly reverses this oxidative stress effect. Additionally, hydrogen peroxide (H_2_O_2_)-induced apoptosis was prevented in PHB-overexpressing cardiomyocytes [52].

It is possible that PHB promotes the mitochondrial membrane permeability transition and inhibits the release of cytochrome c from mitochondria to trigger apoptosis. These studies reflect the protection conferred against oxidative stress-induced apoptosis that has been reported in various mitochondrial complex disorders. These data indicate that PHB protects from oxidative stress-induced damage, and fine tuning of PHB content in mitochondria could be a potential new therapeutic target for myocardial injury and diabetes. Therefore, considering the PHB participation in integral signaling of mitochondrial-mediated cellular disturbance, it is plausible that targeting PHB may inhibit the progression and pathology of diseases characterized by oxidative stress. However, it has also been reported that the treatment of prostate cancer cells with TGF-*β* results in translocation of PHB from the nucleus to the cytosol where it strongly associates with the mitochondrial apoptosis-suppressor Bcl-2 and represses its activity [53]. All these findings suggest that PHB has a diverse array of signaling, and its function may depend on the type of stimulus the cells encounter. Mitochondrial dysfunction in these stressed cells can give rise to a broad spectrum of diseases such as neurodegenerative disorders, cardiomyopathies, optic neuropathy, and inflammatory diseases. During cellular stress, PHB can translocate to the nucleus or to the mitochondria and could possibly trigger apoptotic signals [54]. How a membrane protein such as PHB that can control cell death requires future studies. That is to say, it is possible that PHB not only functions as a transmembrane receptor in the cell but could also be acting as a regulator between cell survival and cell death; although, this is an area that requires further investigation.

## 5. Prohibitin-Ca^2+^ Interface: Possible ER-Mitochondria Ca^2+^ Juxtapose

Positively charged Ca^2+^ ions are one of the primary signaling elements found within cells. In almost all membrane bound organelles, Ca^2+^ binds to hundreds of proteins to impact changes in localization, association, and function [55]. It is also an important second messenger participating in many cellular activities including cell growth, differentiation, gene regulation, and cell survival [56, 57]. Ca^2+^ helps in reversing the damage elicited by cellular insults by both facilitating cell survival and mitigating cell death responses. Interestingly, in the process of adaptive responses, Ca^2+^ participates as a crucial second messenger that decides whether a cell must survive or die. This duality of Ca^2+^ function makes it difficult to delineate mechanisms required for Ca^2+^-mediated control of cell survival and apoptosis. In the pancreatic *β*-cells, it is important to determine the source of the disruption in Ca^2+^ signaling that ultimately affects the overall *β*-cell function. Researchers have also proposed the role of mitochondria in such events; however, its involvement in this process remains poorly understood. One of the most important questions to-date in the field is understanding the role that PHBs might play with ER and mitochondrial Ca^2+^ storage. However, untill now, PHBs are not known to regulate Ca^2+^, but it has been shown that PHB2 regulates the Mg^2+^ channel Transient Receptor Potential Melastatin 6 (TRPM6) [58]. TRPM6 functions as the gatekeeper of transepithelial Mg^2+^ transport and regulation by PHB hints the role of PHBs in the possible interaction with the divalent cationic molecule like Ca^2+^. In this section, we will review the kinetics of Ca^2+^ as an indicator of *β*-cell function and explain the effect of proinflammatory cytokines on Ca^2+^ oscillation in *β*-cells. Cytokines induce a disruption in Ca^2+^ signaling that can impair insulin release in response to glucose stimulation, and in chronic cases, can even lead to *β*-cell death. In particular, IL-1*β* can alter intracellular Ca^2+^ levels by depleting Ca^2+^ from the ER via Ca^2+^ channels such as inositol 1,4,5-trisphosphate receptor (IP_3_R), Ryanodine receptor (RYR), and sarco/endoplasmic reticulum Ca^2+^-ATPase (SERCA) pump.

**Figure 3:**
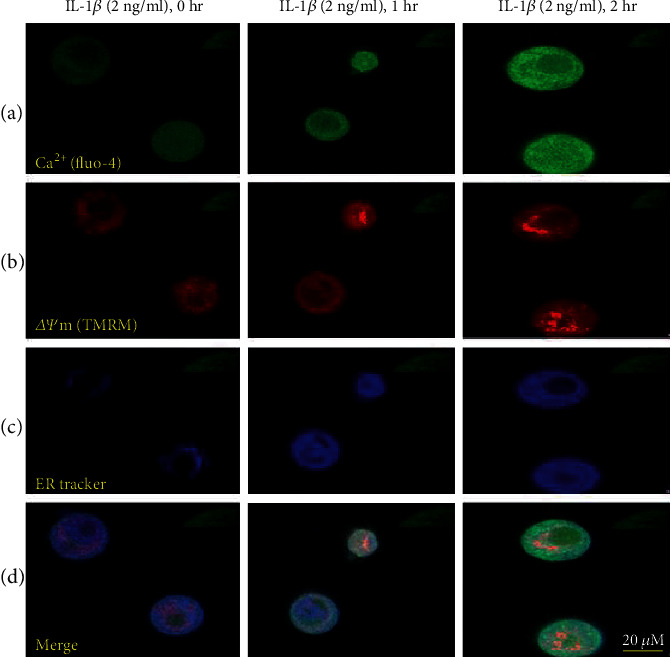
Fluorescent imaging of *β*-cells with Fluo-4, TMRM, and ER tracker. Mice islets were isolated and dissociated in *β*-cells. Further, *β*-cells were induced by IL-1*β* (2 ng/ml) for 0 hr, 1 hr, and 2 hr, respectively, and were processed for Ca^2+^ imaging with respective fluorescent dyes. Panel shows differential expression of Ca^2+^ level in different compartments of the cell. Panel (a) shows the level of cytosolic Ca^2+^as measured by Fluo-4 (green), panel (b) shows the (*ΔΨ*m) with TMRM (red), panel (c) shows the endoplasmic reticulum with ER tracker (blue), and the merged image of all three fluorescence are represented in panel (d). Leica live cell imaging microscope was used to perform the imaging.

These ER Ca^2+^ channels can in turn facilitate the increase in Ca^2+^ flux to the cytosol following its sequestration in cellular organelles such as mitochondria [59]. Sudden elevation of mitochondrial Ca^2+^ (Ca^2+^)_m_ can lead to Ca^2+^ toxicity and trigger apoptosis. It is now widely accepted that depletion of Ca^2+^ levels leads to protein misfolding and activation of the unfolded protein response (UPR) [60]. Organelles such as the ER, mitochondria, and nucleus are also affected by cytokine like IL-1*β* which may lead to *β*-cell death in type 1 diabetes (T1D) and type 2 diabetes (T2D). IL-1*β*-induced changes in the level of the Ca^2+^ in the ER and cytosol, and its resulting impact on mitochondrial physiology like mitochondrial membrane potential (*∆Ψ*m) are depicted in (Figure 3). As evident from (Figure 3), IL-1*β* treatment results in a time-dependent increase in (Ca^2+^)_c_ as measured by Fluo-4 with a concomitant increase in *∆Ψ*m by TMRM. Analysis of the ER in the IL-1*β*-treated cells shows an increase in ER size, suggesting ER attempts to mitigate the effect of IL-1*β* by increasing its biogenesis. It is important to further study Ca^2+^ signaling in these compartments, which could be a worthy area of investigation in both normal and pathological cellular processes.

## 6. Studying Prohibitin-Ca^2+^ Signaling with Live Cell Imaging: A Boon for Ca^2+^ Studies

Using the live-cell imaging technology coupled with focused fluorescent dyes, the effect of proinflammatory cytokines on Ca^2+^ flux in pancreatic *β*-cells has been extensively studied [61, 62]. Numerous reports have implicated Ca^2+^ oscillation as critical in ER and mitochondria to maintain pancreatic physiology [63, 64]. Extensive research demonstrates that Ca^2+^ is confined to particular regions of pancreatic *β*-cells, principally in the ER, and mitochondria [65, 66]. Likewise, observed Ca^2+^ movements from the ER to mitochondria are induced by cytokines that promote mitochondrial dysfunction and cell death [67]. As stated earlier, the role of PHB in controlling mitochondrial structural integrity, and subsequently *β*-cell capacity, has been studied extensively; however, there has been no experimental evidence that suggests the possible role of PHB in the regulation of Ca^2+^ signaling in *β*-cell mitochondria. Given that PHB is a mitochondrial chaperone and could regulate Ca^2+^ in *β*-cell physiology, we hypothesize PHB-mediated regulation of Ca^2+^ could play a role in the *β*-cell function.

## 7. Prohibitin-Ca^2+^ Signaling Crosstalk: Potential Candidate in *β*-Cell Dysfunction

**Figure 4:**
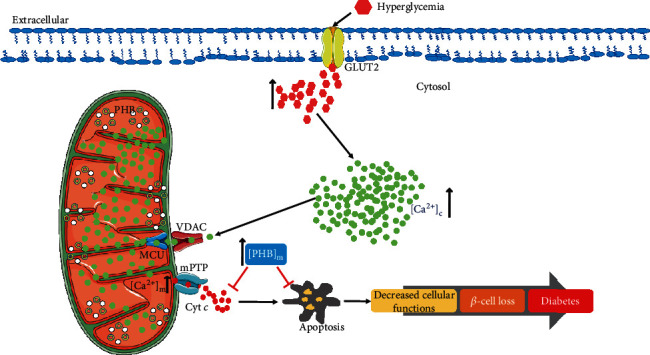
Plausible role of prohibitin in mitochondrial Ca^2+^ overload. During hyperglycemic condition, there is a remarkable increase in cytosolic Ca^2+^ (Ca^2+^)_c_. Physiologically, the elevation of (Ca^2+^)_c_ is required for maintaining the normal metabolic pathways; however, persistent elevation leads to the movement of the (Ca^2+^)_c_ to the mitochondria and the subsequent rise in mitochondrial Ca^2+^ (Ca^2+^)_m_ concentration. Prohibitin may attempt to buffer this excess of (Ca^2+^)_m_ to further prevent the mitochondrial-mediated apoptosis by preventing the release of cytochrome *c* (Cyt *c*). The consequence of this event may lead to decreased cellular function, *β*-cell loss, and diabetes. Overexpression of prohibitin may help in sequestering excess of the Ca^2+^ and prevent apoptosis. Mitochondrial calcium uniporter (MCU) and voltage-dependent anion channel (VDAC) are the channels on the inner and outer surface of mitochondria that sequesters the (Ca^2+^)_m_ cooperatively.

Understanding the role of PHB in Ca^2+^ regulation will aid in the understanding of the mitochondrial capacity and dysfunction in metabolic pathologies like diabetes. Recent reports suggest that *β*-cells from PHB2^−/−^ mice display a varing degree of mitochondrial dysfunction immediately after cell subculture [68]. Cytokine or glucolipotoxicity induced changes in Ca^2+^ levels enhance Ca^2+^ flux out of the ER through ER Ca^2+^ channels, thereby diminishing the Ca^2+^ pool stored in the ER. Cellular stressors like cytokines, oxidative stress, and hyperglycemia impair the storage capacity of the ER, resulting in elevated (Ca^2+^)_c_ and subsequent mitochondrial dysfunction. Given that PHB is a mitochondrial chaperone, it is possible that PHB participates in Ca^2+^ buffering during (Ca^2+^)_c_ elevation. Depletion of PHB could result in adverse effects associated with elevated (Ca^2+^)_c_ levels. The outline of the current hypothesis is depicted in (Graphical Abstract) and in (Figure 4). However, the role of Ca^2+^ in PHB-mediated maintenance of mitochondrial function in pancreatic *β*-cells, and how PHB may mitigate future risk of T2D is not yet explored; additional studies investigating this process are needed to aid in the development of therapeutics for diabetes.

In the following section, experimental approaches to monitor Ca^2+^ flux in *β*-cells and the role PHB plays in regulating Ca^2+^ flux will be discussed. To date, confocal and live cell microscopes are the most commonly used approach to evaluate Ca^2+^ oscillation in live cells. Cellular stressors (cytokines and hyperglycemia) impair Ca^2+^ flux in living *β*-cells, of which the immediate impact on PHB function can be easily monitored with the help of genetically encoded Ca^2+^ indicators (GECI), Ca^2+^ dyes and fluorescence microscopy. These commercially available indicators are widely used to determine localization of Ca^2+^. To accurately measure the Ca^2+^ flux changes in *β*-cells after stress induction, different combinations of fluorophores can be used to identify the subcellular region that exhibits fluorescent emission upon laser stimulations [69, 70]. Fluorescent markers like 4mtD3cpV (mitochondrial FRET-based Ca^2+^ sensor), D1ER [second-generation cameleon (calcium sensor) targeted to ER], and fluo-4/FURA-2AM can be used to evaluate Ca^2+^ oscillations in mitochondria, ER, and cytosol, respectively [71]. In most studies involving diabetes, either a combination of cytokines such as TNF-*α*, IL-1*β*, and IFN*γ* or 15-30 mM glucose mimicking hyperglycemia may be used to stimulate Ca^2+^ release from the ER. Cellular stress results in altered Ca^2+^ signaling thereby promotes mitochondrial dysfunction and ultimately culminate in apoptosis.

Despite the lack of evidence directly linking PHB to Ca^2+^ flux, PHB has been implicated in both *β*-cell and mitochondrial physiology. *β*-cell specific PHB2 knockout mice (*β*-PHB2 ^−/−^) have dysfunctional mitochondria, enhanced *β*-cell death, and diabetes [68]. However, it remains to be observed how this mitochondrial chaperone participates in handling Ca^2+^ toxicity and to what extent the Ca^2+^ burden imposed by the cellular insults is mitigated by PHB? PHB serves as a chaperone ensuring the correct order of the protein translation machinery in the cell. It is therefore imperative to study its role in this phenomenon. Given that the majority of chaperones require Ca^2+^ for their function [72, 73], we hypothesize that PHB could be a plausible candidate in sequestering excess (Ca^2+^)_m_, inhibiting the execution of apoptosis by mitochondria. Primarily, mitochondria sequester the (Ca^2+^)_c_ by inner mitochondrial membrane (IMM) mitochondrial calcium uniporter (MCU) in cooperation with voltage dependent anion channel (VDAC) which is expressed at the outer mitochondrial membrane (OMM) surface (Figure 4). It is also interesting to note that PHBs interact with VDAC [74] and since VDAC is majorly involved in the permeability pathways that control the flow of Ca^2+^, it is very likely that PHBs could regulate the Ca^2+^ signaling both dependent and independent of VDAC. The mitochondrial architecture and its morphology are principally maintained by the coordination of organellar and cytosolic communication. The involvement of Ca^2+^ signaling in the regulation of mitochondrial morphology in physiological and pathological conditions remains to be understood. Nevertheless, there are reports demonstrating Ca^2+^ playing a more central role in orchestrating these processes than previously understood [75].

We, therefore, postulate that excess Ca^2+^ in the mitochondria could be sequestered by overexpression of PHBs, which in turn could prevent the induction of cell death or other pathological conditions. To better understand the role PHB plays in Ca^2+^ signaling, several approaches could be employed. For example, studying ER Ca^2+^ channels in partial knockdown of PHB could help distinguish the role of these channels in Ca^2+^ release.

Overexpression or knockdown of PHBs can reveal the degree to which PHB contributes to mitochondrial Ca^2+^ overburden, and further, delineate the role of PHB in buffering the elevated Ca^2+^. This will aid in understanding the link between PHB and Ca^2+^ homeostasis in cellular physiology. Blocking Ca^2+^ channels using Ca^2+^ channel blockers and monitoring the expression of PHBs would be a simple and valuable experiment in testing the hypothesis that PHB regulates Ca^2+^ exchange between the ER and mitochondria, particularly in *β*-cells. Alternatively, a more significant experiment would be to examine whether different types of PHB ligands regulate Ca^2+^ flux as described by Wang et al. [76]. In the context of Ca^2+^-PHB orchestration, an optional methodology of cameleon test (genetically encoded fluorescent markers for Ca^2+^) could also be utilized for monitoring of Ca^2+^ movement in different cell types [77]. Cameleon tests like D1ER and 4mtD3cpV are very efficient to perform FRET-based imaging to monitor alterations in Ca^2+^ levels induced by cytokines or glucolipotoxicity challenges. Such experiments would help in characterizing the phenomena involving Ca^2+^ levels and PHB capacity to prevent *β*-cell death.

## 8. Role of Prohibitin in Membrane Signaling and Cellular Fate: A Binary Switch?

PHBs are located in various membranes where they play an instrumental role in cellular signaling. PHBs are engaged in the tyrosine phosphorylation PI3K/Akt, MAPK/ERK pathways, and TGF*β* signaling pathways, suggesting the importance of PHB in processes such as metabolism, proliferation, and development [78]. PHB has also been shown to be required for the Ras-mediated Raf-MEK-ERK activation, a critical process that occurs in conjunction with the plasma membrane [79]. PHB can also indirectly facilitate the crosstalk between the PI3K/Akt and Ras/MAPK/ERK pathways mediated by its signaling intermediates PtdIns (3,4,5) *P*_3_, SH2-domain containing phosphatase (Shp)1 [80]. PHB has also been shown to be the intracellular effector molecule of TGF-*β* signaling in prostate cancer cells. Loss of PHB also culminates in apoptosis via the TGF-*β*-Smad axis suggesting its role in cellular apoptosis [53]. PHB levels vary under different stimuli, and it can be said that it acts as both a proapoptotic as well as antiapoptotic molecule, depending upon the type of stress the cell has encountered with. It is reported that oxidative stress induces deleterious effects in pancreatic *β*-cells [81, 82].

However, PHB expression in pancreatic *β*-cells is protective against oxidative stress and apoptosis [83]. Furthermore, we previously reported, IL-1*β*, a pro-inflammatory cytokine induces ER stress and mitochondrial-mediated apoptosis in pancreatic *β*-cells. This action is mediated by JNK, and in part, by Ca^2+^ [59]. In addition, mitochondrial architecture is primarily governed by the integration of organellar and functional protein crosstalk. Mitochondria help in buffering excess (Ca^2+^)_c_ and maintain the appropriate Ca^2+^ level in the cells [84]. During stress, mitochondrial strength is compromised which in turn leads to mitochondrial Ca^2+^ overload and apoptosis in pancreatic *β*-cells. PHB stabilizes mitochondrial proteins by functioning as a chaperone. The majority of chaperones need Ca^2+^ to function [85, 86]. However, the role of Ca^2+^ in regulation of mitochondrial PHB in both normal and pathological conditions is not known. Recent reports have suggested that Ca^2+^ plays a critical role in maintaining mitochondrial health and proper function [87, 88]. Given both PHB and Ca^2+^ are critical players in mitochondrial dynamics, it is possible that a crosstalk exists between PHB and Ca^2+^. PHB, a stabilizer of inner mitochondrial membrane proteins, may participate in Ca^2+^ handling in the mitochondria, thereby preventing mitochondrial Ca^2+^ overload which is a critical instigating event of pancreatic *β*-cell death. In an event of increased (Ca^2+^)_c_, the mitochondria make efforts to buffer elevated (Ca^2+^)_c_, possibly by fine-tuning PHB levels. Such fine-tuning by mitochondria may protect the cell from deleterious events such as altered membrane potential, impaired ATP production, deficiency in insulin secretion, and *β*-cell death. However, the exact role of PHB in handling excess Ca^2+^ in mitochondria is not known. Therefore, PHB may be a potential molecular target that may be manipulated pharmacologically to treat obesity and diabetes. Recent reports involving the role of PHB in obesity draws attention to investigate this axis [89, 90]. Of note, PHB expression has to be modulated physiologically which is otherwise may lead to other serious consequences like obesity and glucose homoeostasis imbalance. It is very interesting to note that almost 25 years ago, McClung et al. [91] speculated prohibitin as an inner mitochondrial membrane protein, which may control ion transport and calcium-dependent ATP production, but surprisingly, there has been no experimental evidence that demonstrates Ca^2+^ regulated PHB function in mitochondria. The possible role of PHB and its role in disease pathology is described in (Table 1).

## 9. Methods Involved in Calcium Signaling: An Era of Modern Biology

Genetically, manipulating PHB expression in both commercially available pancreatic *β*-cells and human islets would provide a more robust model in which PHB function in Ca^2+^ homeostasis may be interrogated. Ca^2+^ signaling may be manipulated intracellularly via commercially available thapsigargin, an ER stress inducer used to discharge Ca^2+^ into the cytosol. To determine how PHBs contribute to Ca^2+^ buffering, mitochondrial targeted GECI like 4mtD3cpV, mtGeCo1, and mt Pericam can be used to monitor the continuous movement of (Ca^2+^)_m_.

The respective fluorescence of these GECI in the mitochondria will delineate the capacity of PHBs to buffer excess (Ca^2+^)_m_. To fully understand these phenomena, experiments performed in *β*-cells PHB2^−/−^ mice (and their control littermate *β*-PHB2^fl/fl^) can be used to provide *in vivo* evidence. Overexpression of PHBs in *β*-cells can aid in evaluating (Ca^2+^)_m_ overload, a hallmark feature in mitochondrial-mediated pancreatic *β*-cell death. Additional analysis using patch-clamp electrophysiology (current and voltage clamps) can also be used to evaluate Ca^2+^ homeostasis as an alternative approach to investigate PHB capacity of buffering excess (Ca^2+^)_c_.

Proinflammatory cytokines, hyperglycemia, and glucolipotoxicity all alter Ca^2+^ levels such that the activated signaling cascades promote pancreatic *β*-cell death during diabetes. However, the mechanisms contributing to the proapoptotic effects of such stressors are not completely understood. In addition to the seminal discovery of the core molecular machinery involved in *β*-cell death, complex Ca^2+^ signaling has emerged as a significant contributor during these events and has gained significant attention. Ca^2+^ actively participates in various physiological processes within pancreatic *β*-cells and rapidly exchanges between the ER and mitochondria. The relationship between the changes in (Ca^2+^)_c_ and (Ca^2+^)_m_ during glucose stimulus was not completely understood. Extensive work has been done in these areas from last two decades to understand this intricate tethering between ER and Mitochondrial Ca^2+^ and their subsequent involvement in ATP regulation and insulin secretion [92–94]. Findings from these studies may provide a foundation upon which future experiments may be designed to identify specific pathways involved in inflammatory or metabolic dysfunction in the *β*-cell. These discoveries illuminate a more focused future on experiments designed to recognize other novel channels involved in Ca^2+^ signaling and mitochondrial dysfunction in *β*-cells. Such novel discoveries also hold promise for unravelling a novel mechanism to aid in the prevention of diabetes.

## 10. Discussion and Future Prospects

Over the years, it has been shown that PHBs interact with other signaling proteins to regulate a multitude of significant cellular events. We do not yet understand the mechanism through which PHBs regulate these events in both healthy and diabetic individuals. In the coming years, one of the biggest challenge's researchers studying diabetes will face is discovering new molecular targets for therapeutic intervention. Identifying the connecting link between PHB expression and Ca^2+^ signaling involved in the aetiology of metabolic diseases would do much to address this challenge and validate PHB as a potential therapeutic target. Nevertheless, emerging roles of mitochondrial-associated ER membranes (MAMs) in the regulation of Ca^2+^ homeostasis cannot be ruled out. MAMs play a crucial role in numerous signaling pathways and are still evolving [95–96]. However, the interaction of PHBs with MAMs has not been studied which could also be a worthy area of investigation.

Studying such interactions may lead to the development of potential drug targets for metabolic diseases. How PHBs influence (Ca^2+^)_c_ and mitochondrial function are still not completely understood. Further studies designed to identify those mechanisms are worthy of investigation and could suggest a role of PHB in maintaining mitochondrial health.

## 11. Conclusions

The goal of this review is to better characterize Ca^2+^ effects on PHBs by directly examining how prohibitin handle Ca^2+^ in the mitochondria during diabetic pathophysiology. We emphasized on the most significant differences observed between control and PHBs knockdown in *β*-cells that should be indicative of the target of Ca^2+^ action, thereby establishing a biomarker for the detection of diabetic phenotype. This will also deliver an additional mechanistic approach to identify the most plausible targets for reversing Ca^2+^ induced damage during diabetes progression. Finally, understanding of this study will determine most substantial effects of PHB_S_ in mitochondrial health and will inform the design of more targeted future experiments to reverse diabetes.

## Figures and Tables

**Table 1:** 

PHB1 function	Location	Subcellular localization	Pathophysiology	Reference
Oxidative stress regulation	*β*-Cells	Mitochondria	Type 2 diabetes	Lee et al., FEBBS J, 2010.
Oxidative stress regulation	Intestinal epithelial cells	Mitochondria	Inflammatory bowel disease	Kathiria e al., Plos One, 2010.
*α*-SMA regulation	Renal cells	Mitochondria	Tubulointerstitial lesions	Zhou et al., J of Recep and Signal Trans, 2013.
Oxidative stress regulation	Cardiomyocytes	Mitochondria	Myocardium damage	Liu et al., Cell Stress Chaperone, 2009.
Oxidative stress regulation	Hepatocytes	Mitochondria	HCV	Ivanov et al., Viruses, 2013.
Combating inflammation	Lungs	Mitochondria	COPD	Soulitzis et al., Resp. Med, 2012.
P-53, Rb tumor regulator	Cancer cells	Nucleus	Cancer	Fusaro et al., JBC, 2003.
Synaptic movement	Neural cells	Mitochondria/nucleus	Schizophrenia	Bemstein et al., Neuromol Med, 2012

## Supplementary Materials

Supplementary materials “Graphical Abstract”.
