# Supplementary material for: A Putative Prohibitin-Calcium Nexus in β-Cell Mitochondria and Diabetes
Source: J Diabetes Res. 2020 Oct 8;2020:7814628. doi: 10.1155/2020/7814628 (PMC7737164; doi:10.1155/2020/7814628)
Supplement: Supplementary materials — “Graphical Abstract”. [file 7814628.f1.pptx]

## Slide 1
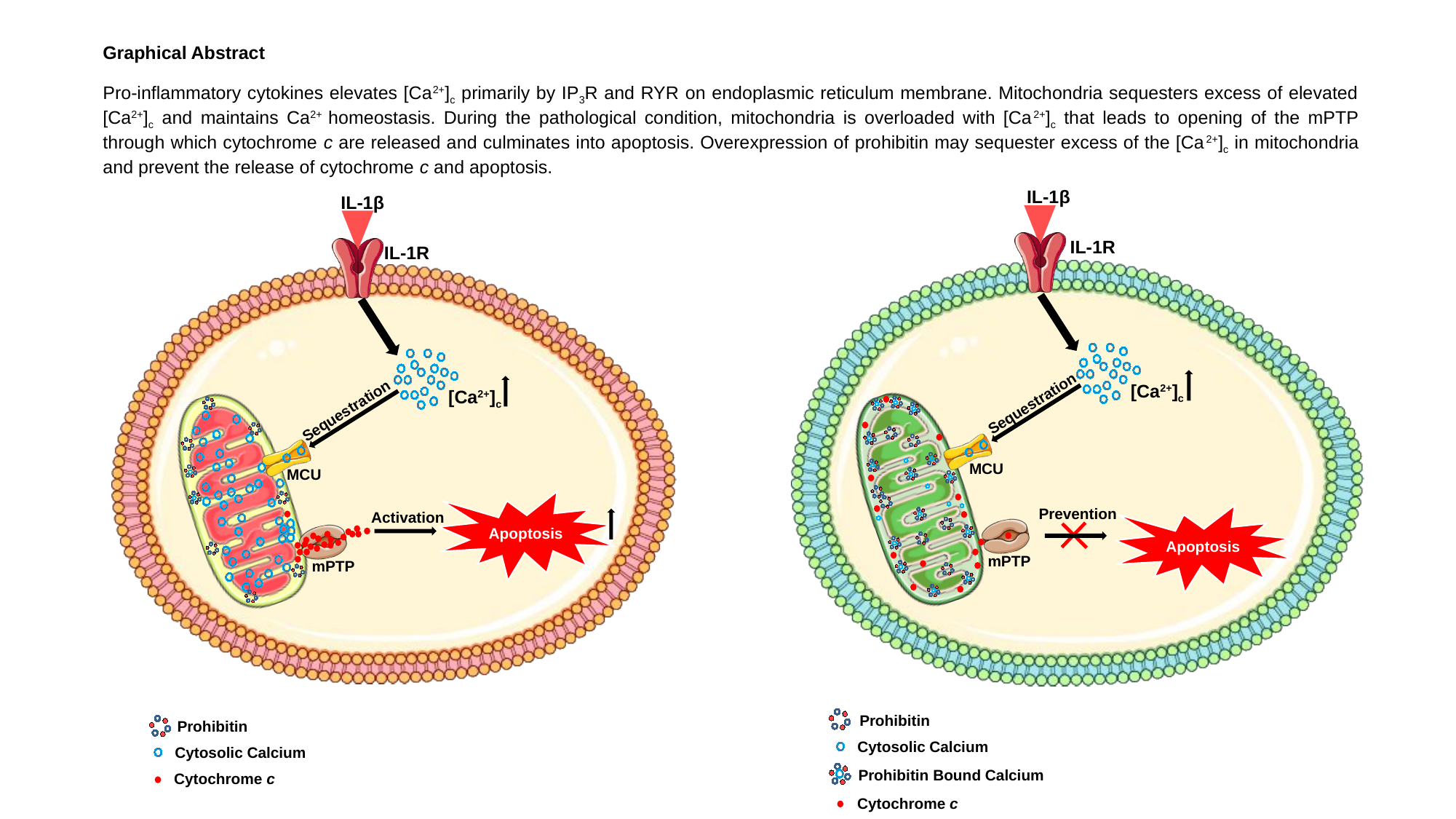

Graphical Abstract
Pro-inflammatory cytokines elevates [Ca2+]c primarily by IP3R and RYR on endoplasmic reticulum membrane. Mitochondria sequesters excess of elevated [Ca2+]c and maintains Ca2+ homeostasis. During the pathological condition, mitochondria is overloaded with [Ca2+]c that leads to opening of the mPTP through which cytochrome c are released and culminates into apoptosis. Overexpression of prohibitin may sequester excess of the [Ca2+]c in mitochondria and prevent the release of cytochrome c and apoptosis.
IL-1β
IL-1β
IL-1R
Sequestration
[Ca2+]c
MCU
Prevention
Apoptosis
mPTP
Prohibitin
Cytosolic Calcium
Prohibitin Bound Calcium
Cytochrome c
IL-1R
Sequestration
[Ca2+]c
MCU
Apoptosis
mPTP
Prohibitin
Cytosolic Calcium
Cytochrome c
Activation
